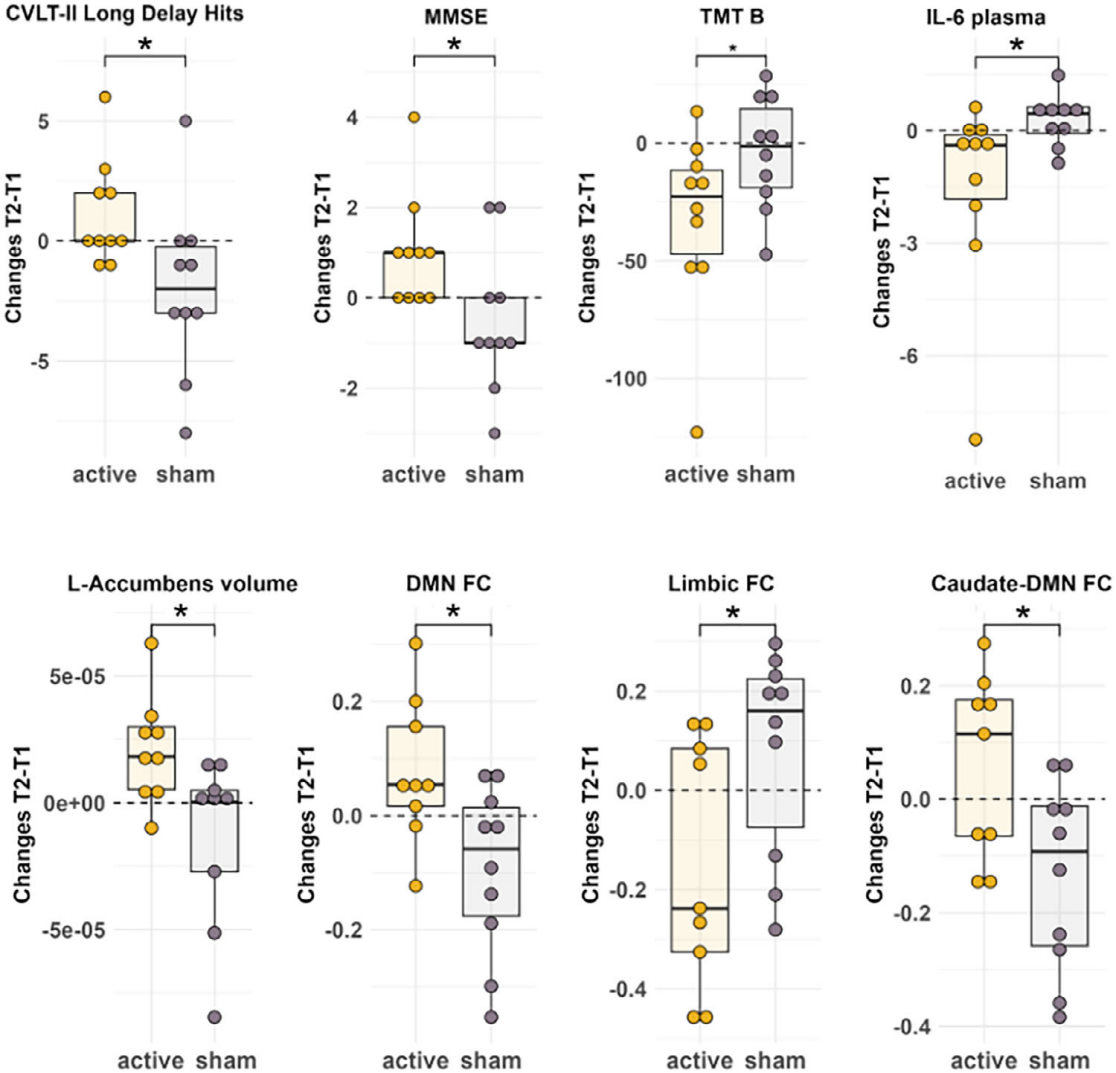# Transcranial Photobiomodulation (tPBM) for Mild Cognitive Impairment (MCI): Key Findings from a Pilot Randomized Clinical Trial (RCT)

**DOI:** 10.1002/alz70861_108308

**Published:** 2025-12-23

**Authors:** Neda Rashidi‐Ranjbar, Nathan W. Churchill, Ana C. Andreazza, Raphael Schneider, Mirjana Jerkic, Ori Rotstein, Reza Zomorrodi, Lew Lim, Simon J. Graham, David G. Munoz, Luis R Fornazzari, Tom A. Schweizer, Corinne E. Fischer

**Affiliations:** ^1^ Keenan Research Centre for Biomedical Science, the Li Ka Shing Knowledge Institute, St. Michael’s Hospital, Toronto, ON Canada; ^2^ Keenan Research Centre for Biomedical Science, Li Ka Shing Knowledge Institute, St. Michael’s Hospital, Toronto, ON, Canada, Toronto, ON Canada; ^3^ Temerty Faculty of Medicine, Department of Psychiatry, University of Toronto, Toronto, ON Canada; ^4^ Department of Pharmacology & Toxicology, Mitochondrial Innovation Initiative, University of Toronto, Toronto, ON Canada; ^5^ Temerty Faculty of Medicine, Division of Neurology, University of Toronto, Toronto, ON Canada; ^6^ Keenan Research Centre for Biomedical Science, Li Ka Shing Knowledge Institute, St. Michael's Hospital, Toronto, ON Canada; ^7^ Temerty Faculty of Medicine, Department of Surgery, University of Toronto, Toronto, ON Canada; ^8^ Temerty Centre for Therapeutic Brain Intervention, CAMH, Toronto, ON Canada; ^9^ Vielight Inc., Toronto, ON Canada; ^10^ Department of Medical Biophysics, University of Toronto, Toronto, ON Canada; ^11^ Hurvitz Brain Sciences Program, Sunnybrook Research Institute, Toronto, ON Canada; ^12^ Temerty Faculty of Medicine, Department of Laboratory Medicine and Pathobiology, University of Toronto, Toronto, ON Canada; ^13^ Keenan Research Centre for Biomedical Science, Li Ka Shing Knowledge Institute, St. Michael’s Hospital, Toronto, ON Canada; ^14^ Temerty Faculty of Medicine, Division of Neurosurgery, University of Toronto, Toronto, ON Canada

## Abstract

**Background:**

Mild Cognitive Impairment (MCI) is a frequent precursor to Alzheimer’s dementia (AD). Mitochondrial dysfunction, marked by reduced cytochrome c oxidase (CCO) activity and lower ATP production, is linked to these neurodegenerative diseases. This study evaluates transcranial photobiomodulation (tPBM), a non‐invasive technique using near‐infrared light to stimulate mitochondrial CCO, potentially enhancing neuronal energy and cognitive function in individuals with MCI.

**Method:**

Twenty patients with mild cognitive impairment (MCI) were randomly assigned to an active treatment group (n = 10) or a sham control group (n = 10) using visually identical devices to maintain blinding. Participants completed daily home‐based tPBM sessions for 6 weeks. Pre‐ and post‐treatment assessments included cognitive tests (MMSE, TMT‐A & B, CVLT‐II) and biomarker evaluations (blood samples via ELISA). Neuroimaging included proton magnetic resonance spectroscopy (¹H‐MRS) of the posterior cingulate cortex (PCC) and whole‐brain structural and resting‐state functional MRI. Change scores were calculated by subtracting baseline from post‐treatment values.

**Result:**

Compliance exceeded 98% in both groups, and tPBM was well‐tolerated. The active tPBM group showed significantly greater post‐treatment improvements from baseline (p < 0.05) compared to the sham group, including: (1) better recognition memory (higher long‐delay hits, fewer false positives on the CVLT‐II); (2) improved cognition (higher MMSE); (3) faster processing speed (shorter TMT‐B times); (4) decreased plasma IL‐6 levels; (5) higher choline/creatine ratio and a trend toward increased myoinositol/creatine in the PCC; (6) increased left nucleus‐accumbens volume; and (7) enhanced functional connectivity within the DMN and between the caudate and DMN, along with decreased FC within the limbic network.

**Conclusion:**

Daily, home‐based tPBM is a well‐tolerated and feasible intervention that led to significant improvements in both cognitive function and biological markers in individuals with MCI. The active tPBM group demonstrated enhanced cognition, recognition memory, and processing speed, alongside reductions in inflammation and structural and functional brain changes, including increased neuroplasticity and alterations in brain connectivity. These findings suggest that tPBM may promote neuronal function and brain network modifications, particularly within the default mode network and limbic regions, providing evidence for its potential as a therapeutic approach in the early stages of Alzheimer's disease.